# Hypoxia and Alpha-Synuclein: Inextricable Link Underlying the Pathologic Progression of Parkinson's Disease

**DOI:** 10.3389/fnagi.2022.919343

**Published:** 2022-07-26

**Authors:** Mengyuan Guo, Xunming Ji, Jia Liu

**Affiliations:** ^1^Beijing Institute of Brain Disorders, Laboratory of Brain Disorders, Ministry of Science and Technology, Collaborative Innovation Center for Brain Disorders, Beijing Advanced Innovation Center for Big Data-based Precision Medicine, Capital Medical University, Beijing, China; ^2^Department of Neurosurgery, Xuanwu Hospital, Capital Medical University, Beijing, China

**Keywords:** alpha-synuclein, hypoxia, oxygen intake, oxygen utilization, Parkinson's disease

## Abstract

Parkinson's disease (PD) is the second most common neurodegenerative disease after Alzheimer's disease, with typical motor symptoms as the main clinical manifestations. At present, there are about 10 million patients with PD in the world, and its comorbidities and complications are numerous and incurable. Therefore, it is particularly important to explore the pathogenesis of PD and find possible therapeutic targets. Because the etiology of PD is complex, involving genes, environment, and aging, finding common factors is the key to identifying intervention targets. Hypoxia is ubiquitous in the natural environment and disease states, and it is considered to be closely related to the etiology of PD. Despite research showing that hypoxia increases the expression and aggregation of alpha-synuclein (α-syn), the most important pathogenic protein, there is still a lack of systematic studies on the role of hypoxia in α-syn pathology and PD pathogenesis. Considering that hypoxia is inextricably linked with various causes of PD, hypoxia may be a co-participant in many aspects of the PD pathologic process. In this review, we describe the risk factors for PD, and we discuss the possible role of hypoxia in inducing PD pathology by these risk factors. Furthermore, we attribute the pathological changes caused by PD etiology to oxygen uptake disorder and oxygen utilization disorder, thus emphasizing the possibility of hypoxia as a critical link in initiating or promoting α-syn pathology and PD pathogenesis. Our study provides novel insight for exploring the pathogenesis and therapeutic targets of PD.

## Introduction

Parkinson's disease (PD) is the second most common neurodegenerative disease of the central nervous system (Tolosa et al., 2021). The typical pathological characteristics of PD are the progressive degeneration and loss of dopaminergic neurons in the substantia nigra, resulting in dopamine deficiency and the formation of Lewy bodies (LBs) in the remaining neurons, the main component of which is alpha-synuclein (α-syn) (Dunn et al., 2019). Epidemiological studies show that the current incidence of PD is about 1% to 2% in people over the age of 65 years. It is roughly estimated that there are 7–10 million patients with PD worldwide (Aarsland et al., 2021). Over the last 30 years, the number of patients has increased 2.5 times. Meanwhile, its prevalence is expected to double over the next 30 years as the population ages (Dorsey et al., 2018b). In addition, with the rapid development of modern industry and inevitable environmental pollution and other factors, PD may even usher as a new pandemic and bring a heavier social burden (Bloem et al., 2021).

Unfortunately, the etiology of PD is complex, the pathogenesis is unknown, and there is no effective treatment. As for the etiology, genes, environment, aging, traumatic brain injury (TBI), and poisoning are recognized as major risk factors (Klein and Westenberger, 2012; Dunn et al., 2019). From the perspective of the pathological mechanism, PD is inseparable from mitochondrial dysfunction (Bose and Beal, 2016), autophagy-lysosome disorder (Lu et al., 2020), vesicular transport disorder (Nguyen et al., 2019), and neuroinflammation (Marogianni et al., 2020). α-syn is the major component of LBs, playing a central role in PD induced by various risk factors, and SNCA encoding this protein is the first PD risk gene to be discovered (Polymeropoulos et al., 1997). α-syn is a protein widely expressed in the brain, with strong physiological functions. There are several important stages in the transformation of α-syn, from playing physiological functions to promoting pathology, such as phosphorylation modification, aggregation (Tu et al., 2021), and propagation (Garcia et al., 2022), thus promoting the development of PD. Due to the complex risk factors and pathogenesis mentioned above, PD intervention is challenging. We sought to find common ground among the many risk factors and intricate pathogenesis of PD. We were surprised to find that oxygen intake and utilization disorders seem to link the pathogenesis and pathology of PD. Whether the source is environmental hypoxia or tissue hypoxia, we can collectively refer to this key link as hypoxia.

This review summarizes the risk factors of PD, including genes, environment, aging, and TBI. Further, we discuss the relationship between oxygen intake disorder, oxygen utilization disorder, and the above risk factors. We describe the transformation process of α-syn in PD pathogenesis, including phosphorylation modification, aggregation, and propagation, and highlight the important role of hypoxia in promoting α-syn pathology. This review aims to interpret the role of hypoxia in the development of PD from a new perspective, so as to find a creative breakthrough for the defense and treatment of PD.

## Risk Factors for Parkinson's Disease

The etiology of PD is unknown. It is not a single-factor disease but rather one characterized by a combination of factors (Elbaz et al., 2016). Specifically, the main risk factors for PD include genes (Blauwendraat et al., 2020), environment (Murata et al., 2022), aging (Pang et al., 2019), and TBI (Cruz-Haces et al., 2017). In what follows, we summarize these risk factors in order to find the possible common links among them.

### Genes

So far, more than 20 genes have been found to have risk variants associated with PD, including rare genetic variants and common genetic variants. Seven of the most widely studied genes are SNCA, LRRK2, PINK1, PARK2, DJ-1, VPS35, and ATP13A2 (Dunn et al., 2019; Blauwendraat et al., 2020). SNCA was the first reported PD-related pathogenic gene (Polymeropoulos et al., 1997). PD can be induced by missense mutations and genomic multiplications in SNCA (Pihlstrøm and Toft, 2011). In the same year, α-syn encoded by SNCA was reported to be the main component of LBs (Spillantini et al., 1997, 1998). A series of subsequent studies established the stable core position of α-syn in the PD process. Considering the important role of α-syn in the pathogenesis of PD, α-syn pathology is introduced and discussed in detail in the third part of this review.

### Environmental Risk Factors

According to epidemiological reports, the prevalence of PD is biased by region and race, suggesting the importance of environmental factors to some extent (Pringsheim et al., 2014). The incidence of PD is directly proportional to the urbanization process and the speed of industrial development. This may be because economic growth represents more environmental pollution, such as air pollution, heavy metals, pesticides, and neurotoxic chemicals (Dorsey et al., 2018a,b; Bloem et al., 2021).

#### Air Pollution

Exposure to air pollution increases the risk of PD (Murata et al., 2022). Air pollution includes common atmospheric particulates and toxic gases. The former mainly comes from industrial combustion and exhaust from vehicles powered by diesel fuel (Costa et al., 2020), such as PM2.5. The latter mainly come from industrial boilers and motor vehicles, including carbon monoxide (CO) and nitrogen dioxide (NO_2_). Many studies have proved that air pollution has a negative impact on the central nervous system, which is related to oxidative stress, neuroinflammation, and neurodegeneration (Babadjouni et al., 2017; Thomson, 2019). Exposure to PM2.5 and PM10 was positively associated with PD (Palacios, 2017). For PM10, the risk of PD to those exposed to more than 65 μg/m^3^ was more than 1.35 times higher than those exposed to <54 μg/m^3^ (Chen et al., 2017). Exposure to higher NO_2_ concentrations was associated with a 1.41-fold increased risk of PD relative to the lowest quartile concentration (Jo et al., 2021). In one clinical report, 242 patients with CO poisoning admitted to the hospital were followed up over a 10-year period. Up to 9.5% of them suffered from PD, and most developed PD quickly, with an average age of 45.8 years (Choi, 2002). A systematic review using meta-analysis reported that long-term exposure to CO and nitrogen oxides was positively associated with an increased risk of PD. The prevalence of PD after CO exposure can be as high as 1.65 times (Hu et al., 2019). In addition, PD mainly affects people over the age of 65 years, and the aging brain is very sensitive to air pollution (Costa et al., 2020), which further illustrates the importance of air pollution in the pathogenesis of PD.

#### Pesticides Exposure

Epidemiological studies have shown that people working in agriculture have a higher prevalence of PD, and the results are consistent in rural areas with increased pesticide exposure. There is growing evidence that exposure to pesticides or solvents is a risk factor for PD (Pezzoli and Cereda, 2013; Pouchieu et al., 2018; Tomenson and Campbell, 2021). Pesticides include insecticides and herbicides. Insecticides are divided into organophosphates and organochlorine pesticides. The former is represented by rotenone, while the latter most commonly include dieldrin. The most common herbicide is paraquat (Ball et al., 2019). Neurotoxicity induced by dieldrin is associated with PD (Kanthasamy et al., 2005). Back in 1985, rotenone was used to mimic PD and was shown to cause fatal damage to dopaminergic neurons (Heikkila et al., 1985). Paraquat has a striking similarity in composition to a toxic metabolite of a nerve agent named 1-methyl-4-phenyl-1,2,3,6-tetrahydropyridine (MPTP). In 1983, a study in the journal *Science* reported a correlation between MPTP and PD (Langston et al., 1983). Currently, MPTP is recognized as the classical PD modeling method (Langston, 2017).

#### Heavy Metals

Exposure to heavy metals has attracted ample attention in PD studies. Heavy metals are also considered neurotoxins, causing oxidative stress that can lead to neuronal death (Wei et al., 2020). Common heavy metals include iron (Fe), copper (Cu), manganese (Mn), lead (Pb), and mercury (Hg). Studies have shown increased Fe concentrations in the substantia nigra in patients with PD (Dexter et al., 1987; Wypijewska et al., 2010). It is associated with the progressive degeneration of dopaminergic neurons in the substantia nigra in patients with PD (Devos et al., 2020). Infants who consumed milk powder with a higher iron content had an increased risk of neurodegeneration later in life (Hare et al., 2015). Occupational exposure to Cu increases the risk of PD by more than 2-fold (Gorell et al., 1997, 1999). Long-term inhalation of Mn particles in industrial production, mining, welding, and other activities can cause serious damage to the nervous system and amplify the risk of PD (Caudle et al., 2012; Ullah et al., 2021). Occupational exposure to Pb increases the concentration of Pb in the body and increases the risk of PD by more than 2 times (Coon et al., 2006; Weisskopf et al., 2010). Hg is a neurotoxin that causes neuronal death and can cause movement disorders (Fernandes Azevedo et al., 2012). The report, which focuses on developing countries, found that Hg poisoning can increase the incidence of PD by more than eight times (Ullah et al., 2021).

### Aging

PD tends to occur in the elderly. Epidemiological surveys show that the incidence of PD is low before the age of 50 years, and the average age of PD is about 60 years old. After 65 years of age, the prevalence of the disease increases dramatically, and can even increase 5 to 10 times (Poewe et al., 2017). This undoubtedly establishes the important position of aging in PD pathogenesis (Wyss-Coray, 2016; Hou et al., 2019). With aging, various organs of the body functionally decline, sometimes to the point of dysfunction. An article published in 2013 reviewed nine markers of aging, which were grouped into three broad categories: primary, antagonistic, and integrative hallmarks (López-Otín et al., 2013). Genomic instability, epigenetic alterations, loss of proteostasis, and telomere attrition are primary hallmarks that help cause body damage. As the name suggests, antagonistic hallmarks help combat aging damage, including cellular senescence, deregulated nutrient sensing, and mitochondrial dysfunction. Moreover, altered intercellular communication and stem-cell exhaustion are integrative hallmarks. Integrative hallmarks lead to the ultimate senescence phenotype, with serious consequences, such as organ decline (Aunan et al., 2016; Farr and Almeida, 2018). Notably, almost all hallmarks have been reported to affect the pathologic progression of PD (Hou et al., 2019).

### Traumatic Brain Injury

TBI affects a large number of people, with more than 42 million people suffering from mild TBI each year (Gardner and Yaffe, 2015; James et al., 2019). TBI has been shown to be associated with serious consequences, including neurodegenerative disease and psychiatric sequelae (Wilson et al., 2017). Repeated traumatic injuries can lead to chronic traumatic encephalopathy. According to the study, the increased incidence of PD in retired boxers was positively correlated with their number of professional fights (Bhidayasiri et al., 2012). Boxers and professional football players who receive repeated blows to the head are more likely to suffer motor and behavioral impairments (Yi et al., 2013). In order to eliminate as much interference as possible from lifestyle and experience, twins were included in a study on the association between TBI and PD. It turned out that TBI increased the risk of PD decades later (Goldman et al., 2006). A retrospective cohort of 12 years of follow-up showed that prior TBI increases the risk of PD, and the risk was positively correlated with the degree of injury (Gardner et al., 2018). TBI leads to direct focal lesions such as intracerebral hemorrhage, and diffuse injuries such as hypoxic-ischemic brain injury and vascular injury. The biggest characteristic of TBI is ischemia and hypoxia, as well as obvious inflammatory responses accompanied by oxidative stress and neuron death (Gaetz, 2004; Burda et al., 2016; Yu et al., 2021). This may be the main reason why secondary brain injury increases the risk of PD.

### Stroke

Stroke is one of the leading causes of disability and death worldwide, with ischemic stroke accounting for more than 80% of all patients having stroke (Li W. et al., 2018). As with PD, the elder is at high risk for stroke, with more than 70% of patients over age 60 (Campbell, 2017; Maida et al., 2020). Ischemic stroke leads to a cascade of harmful events, resulting in a lack of nutrients and oxygen in the brain tissue and rapid inflammatory response in the damaged area (Kuczynski et al., 2019). Oxidative stress, mitochondrial dysfunction, autophagy imbalance, abnormal activation of pro-inflammatory factors, and other mechanisms cause neuronal death and neurological dysfunction (Al-Kuraishy et al., 2020; Pluta et al., 2021). Interestingly, oxidative stress and other molecular mechanisms also play a key role in the pathologic process of PD. The abnormal inflammatory response can lead to secondary PD in stroke patients (Rodriguez-Grande et al., 2013). Epidemiological studies have shown that ischemic stroke increases the risk of PD (Lohmann et al., 2022). A prospective cohort of 503,497 volunteers with a mean follow-up of 9 years was used to analyze the association between stroke and PD risk. After accounting for confounding factors such as gender and region, the study reported a 2-fold increase in the risk of PD among those who had previously had a stroke (Kizza et al., 2019). There was a study showed that cerebral ischemia can aggravate PD (Zambito Marsala et al., 2016). Even asymptomatic stroke can worsen the clinical presentation of patients with PD (Nanhoe-Mahabier et al., 2009). It has been demonstrated in animal experiments that after middle cerebral artery occlusion, an asymptomatic stroke occurs in animals, followed by ischemia and neuronal damage in the substantia nigra region, leading to PD (Rodriguez-Grande et al., 2013). It is worth noting that the cellular environment after stroke is dominated by inflammatory activation and oxidative stress, which provides better conditions for abnormal accumulation of protein α -syn. α -syn, as a key pathological protein of PD, is also expressed at a high level in stroke patients, and its ability to form oligomers is enhanced to play a harmful role (Zhao et al., 2016). Parkin and PINK1, which are closely related to PD, are also involved in neuronal death after ischemic stroke injury (Kim and Vemuganti, 2017).

### Others

PD is considered a complex multifactorial disease. In addition to the above factors, vitamin D deficiency (Newmark and Newmark, 2007), high dietary fat intake (Qu et al., 2019), dairy intake (Hughes et al., 2017), gender (Lubomski et al., 2014), race (Wright Willis et al., 2010), drug abuse (Mursaleen and Stamford, 2016), infection (Kline et al., 2021), and other factors may increase the risk of PD. In addition to single-level factor analyses, multifactor interactions should be noted.

## Relationship Between Hypoxia and PD Risk Factors

Genes, environment, aging, and TBI have currently been recognized as PD risk factors, and even multifactor interactions exist in the vast majority of patients. Interestingly, we found that oxygen intake and oxygen utilization disorders were prevalent among the above factors. Damage associated with risk genes often induces mitochondrial dysfunction, resulting in oxygen utilization disorders. Environmental pollution can affect oxygen intake through inadequate ventilation. CO poisoning results in competitive hemoglobin binding and, consequently, oxygen utilization disorders in cells. Aging is accompanied by decreased oxygen utilization in multiple organs. TBI can cause local ischemia and hypoxia in tissues. Therefore, in the following, we discuss the relationship between hypoxia and PD risk factors from the perspectives of insufficient oxygen intake and oxygen utilization disorders.

### Hypoxia and Hypoxia Response

Hypoxia occurs when oxygen levels in local or systemic tissues decrease and are insufficient to maintain normal metabolism, sometimes even making it difficult to survive (Yeo, 2019). Hypoxia is one of the most common stressors, whether from environmental hypoxia or body hypoxia. Environmental hypoxia is common in the plateau, diving, and aviation. For example, most people's hemoglobin oxygen saturation drops at altitudes above 2,500 meters, where more than 140 million people now live (Bigham and Lee, 2014). Hypoxia can be seen in a variety of diseases, including ischemic/hypoxic disease, pulmonary hypertension, atherosclerosis, and cardiovascular diseases such as heart failure (Semenza et al., 2000; Liu et al., 2020). It is worth mentioning that with the increased age, the body and tissues also suffer from a certain degree of hypoxia, especially in the brain, an organ with high oxygen consumption, so it is more urgent to explore the relationship between hypoxia and neurological diseases (Correia et al., 2013).

Heart, lung, and skeletal muscle are the main organs for oxygen delivery and utilization (Strasser and Burtscher, 2018), aging is associated with a decrease in maximum oxygen utilization (Betik and Hepple, 2008). The organ we care most about is the brain, which accounts for only about 2% of our body weight but consumes a fifth of our oxygen. The ability of nerve tissue to use oxygen decreases in an age-dependent manner, and brain tissue requires more oxygen to meet its actual needs (Catchlove et al., 2018). The cerebral metabolic rate of oxygen (CMRO_2_), a measure of brain energy homeostasis, is reduced in the elderly (Zhang et al., 2010). At the same time, the baseline of cerebral blood flow (CBF) decreases in age dependence, and the imbalance between supply and demand also makes it difficult to maintain normal oxygen balance in the brain (Ances et al., 2009).

When faced with hypoxic stress, the body and cells adopt a series of response mechanisms. For the body, increased erythropoiesis, hemoglobin content, and new blood-vessel formation are important measures of hypoxia response (Catrina and Zheng, 2021). The discovery that hypoxia-inducible factors (HIFs) mediate the response to intracellular hypoxia is currently recognized as the most critical link. This discovery was awarded the 2019 Nobel Prize in Physiology or Medicine. HIFs are heterodimeric transcription factors composed of alpha-subunits and beta-subunits. There are three α-subunits—HIF-1α, HIF-2α, and HIF-3α–and two β-subunits, named HIIF-β and ARNT2. The regulation of α-subunits is oxygen-dependent. To ensure normal bodily homeostasis, HIF is strictly controlled by oxygen sensors. Under normoxic conditions, the body does not need the accumulation of HIFs in the body, and the α-subunits are targeted for degradation (Li et al., 2020). However, under hypoxia conditions, α-subunits are stable and bind to constitutive β-subunits, forming a basic helix-loop-helix-PAS domain transcription factor, named HIF. HIF helps activate a series of target genes that regulate cell movement, angiogenesis, red blood cells, hemoglobin, and energy metabolism, helping the body adapt to hypoxia (Corrado and Fontana, 2020). HIF-1α plays an important role in PD. Downstream target genes of HIF-1α, such as EPO and VEGF, have been proved to protect neurons from injury in PD (Zhang et al., 2011). HIF-1α activates a variety of transcriptional processes and targets oxidative stress, such as autophagy, mitochondrial function, and other pathways, which affect PD development. Therefore, HIF-1α has become a potential drug intervention target for PD (Lestón Pinilla et al., 2021).

HIF-1α is a major transcription factor in response to hypoxia. Current studies suggest that HIF-1α plays an important role in the complex pathogenesis of PD. Under normal physiological conditions, prolyl hydroxylases (PHD) contribute to HIF-1α degradation to maintain homeostasis. Currently, PHD inhibitors are believed to increase HIF-1α expression and play a neuroprotective role (Lee et al., 2009). FG-4592 is a PHD inhibitor that effectively reverses MPP^+^ induced cytotoxicity and apoptosis. Meanwhile, FG-4592 treatment 5 days in advance alleviated the damage of dopaminergic neurons in MPTP-PD mice, thus alleviating behavioral disorders (Li X. et al., 2018). The cellular model of PD was established by exposing SH-SY5Y cells to 6-hydroxydopamine (6-OHDA), and Hydralazine preconditioning upregulated HIF-1α, improved TH protein expression, and rescued cell damage (Mehrabani et al., 2020). In the rotenone-induced *in vitro* PD model, agmatine treatment upregulated HIF-1α and effectively alleviated cell damage. However, the presence of HIF-1α inhibitor methyl 3-[[2-[4-(2-adamantyl) phenoxy] acetyl] amino]-4-hydroxybenzoate blocks this protective effect (Ferlazzo et al., 2020). As mentioned above, both activation and inhibition of HIF-1α affect PD, and more importantly, HIF-1α upregulation is negatively associated with neuronal injury in PD. As a key hypoxic response molecule, the above conclusion concerning HIF-1α seems to be inconsistent with the involvement of hypoxia in inducing PD abnormal pathology. However, hypoxia is complex, causing multiple damages and maybe insufficient HIF response ability. The role of HIF-1α in PD is not equivalent to the relationship between hypoxia and PD, which needs to be further explored.

### Risk Factors and Insufficient Oxygen Intake

From the point of view of pathophysiology, hypoxia includes hypoxic hypoxia, hemic hypoxia, and circulatory hypoxia. Decreasing oxygen partial pressure in inhaled gas, pulmonary ventilation dysfunction, and venous blood shunt into the artery leading to hypoxic hypoxia. Abnormal hemoglobin content, structure, and function can cause hemic hypoxia. Circulatory hypoxia is mainly caused by local or systemic circulatory dysfunction.

Air pollution can cause endothelial dysfunction, vasoconstriction, and diseases of the respiratory and cardiovascular systems, resulting in circulatory hypoxia caused by blood supply disorders and hypoxic hypoxia caused by a deficiency in ventilation (Wauters et al., 2015). Particulate air pollution and harmful gases such as sulfur dioxide (SO_2_), in addition to increasing the risk of respiratory and cardiovascular diseases, have also been linked to hypoxic hypoxia caused by reduced oxygen saturation (Luttmann-Gibson et al., 2014). Studies have found that exposure to automobile exhaust pollution triggers the HIF-1 response pathway and ultimately leads to disease. HIF-1 is the key factor in hypoxia response, which also adds supplementary evidence for the relationship between air pollution and hypoxia (Liang et al., 2021; Wu et al., 2021). Some studies used 10% O_2_ hypoxia exposure as a positive control to trigger disease phenotype, and automobile exhaust, one of the main sources of urban air pollution, as a trigger factor in mouse models. The results were consistent with 10% O_2_ hypoxia exposure. This also proves the connection between air pollution and hypoxia in a certain sense (Liu et al., 2018). Compared with oxygen, CO is more likely to bind to hemoglobin and convert oxyhemoglobin to carboxyhemoglobin, so its main toxic mechanism is hypoxia (Horner, 2000; Lacerda et al., 2005). CO causes hemic hypoxia and can even induce hypoxia in fetuses *in utero* (Ion and Bernal, 2015). Intrauterine hypoxia from air pollution is directly or indirectly linked to future brain development and function (Fajersztajn and Veras, 2017).

The aging process is accompanied by vascular damage and vascular aging, so heart failure and other cardiovascular diseases occur easily (Katsuumi et al., 2018). The typical pathophysiological process of heart failure, coronary heart disease, ischemic stroke, and other diseases that the elderly are prone to suffer from is ischemia and hypoxia (Meng et al., 2017). Aging individuals are at increased risk of cardiac ischemia and are more prone to ischemia-reperfusion injury than adults (Ham and Raju, 2017). The above belongs to circulatory hypoxia. Respiratory diseases frequently occurring in the elderly, such as chronic obstructive pulmonary disease (COPD), are mostly related to systemic or local hypoxia (Bradley et al., 2021), which belongs to hypoxic hypoxia.

Hypoxia of the brain is a common secondary injury in patients after TBI. In patients with severe TBI, up to 45% of patients have hypoxia (Thelin, 2016). Hypoxia leads to worse clinical outcomes, setting off a vicious cycle (Yang et al., 2013). Hypoxia is not necessarily ischemia, but ischemia is always accompanied by tissue hypoxia. Traumatic bleeding can lead to systemic ischemia. Intracranial hemorrhage often occurs in patients with TBI, followed by increased intracranial pressure and decreased cerebral perfusion pressure leading to ischemia. The model of traumatic bleeding has been widely used to study ischemia and hypoxia (Ham and Raju, 2017). This is often associated with circulatory hypoxia.

### Risk Factors and Oxygen Utilization Disorders

Oxygen utilization disorders, also known as tissue hypoxia, result from a decrease in the ability of cells and tissues to use oxygen. Tissue hypoxia can be caused by mitochondrial damage or function inhibition and decreased respiratory enzyme synthesis. Oxidative phosphorylation is the main pathway of ATP production, and its main substrate is O_2_. Mitochondria is the main site of oxidative phosphorylation. Therefore, any factors that affect mitochondrial respiration or oxidative phosphorylation may cause oxygen utilization disorders. Respiration substrates oxidize in the mitochondrial matrix to produce NADH and FADH_2_. With the help of electron and hydrogen carriers, they transfer protons and electrons to O_2_ and form water. The electron transport system composed of carriers is called the electron transport chain because the transport chain is directly related to respiration. Thus, it is called the mitochondrial respiratory chain. The respiratory chain is made up of four mitochondrial complexes, and inhibition of any link results in impaired respiratory function.

Although there is no evidence that PD-related genes directly cause hypoxia, induction of mitochondrial dysfunction is the main pathway of most of the pathogenic mechanisms involved in genes, which can directly cause oxygen utilization disorders. Overexpression of α-syn can cause mitochondrial rupture, mitochondrial membrane permeability change, and mitochondrial function impairment, ultimately leading to decreased respiratory function and neuronal death (Nakamura et al., 2011; Shen et al., 2014). α-syn oligomers and aggregates interact with mitochondrial outer-membrane substrates to induce mitochondrial dysfunction. In addition, α-syn can also impair autophagy, including mitochondrial autophagy (mitophagy), and dysfunctional mitochondria cannot be cleared, further exacerbating the damage. PARK2 mutation is the most frequent recessive inheritance in PD, which can reach more than 70% in familial early-onset PD, followed by PINK1 mutation, accounting for about 9%. PINK1 and Parkin mediate a variety of pathways that regulate mitophagy and play an important role in the process of mitophagy (Kilarski et al., 2012; Klein and Westenberger, 2012). Both mutations can cause mitophagy defects (Pryde et al., 2016; Li et al., 2017). DJ-1 is also crucial to mitochondrial function and can act as a redox sensor in mitochondria. Abnormal expression of DJ-1 can lead to mitochondrial defects and increase oxidative stress, affecting mitophagy. Heterozygous GBA1 mutation is a common genetic risk factor for PD and can lead to the aggregation of α-syn and participate in mitochondrial dysfunction, increasing the risk of PD by more than 20 times (Liu et al., 2019). VPS35 mutation can lead to mitochondrial dysfunction, α-syn accumulation, and increased reactive oxygen species (ROS) (Tang et al., 2015; Wang et al., 2016). It has been confirmed *in vitro* and *in vivo* that VPS35 leads to extensive mitochondrial rupture and inevitable functional defects (Wang et al., 2016). There is also evidence that LRRK2 mutations increase mitophagy and affect normal function (Yakhine-Diop et al., 2019). Existing studies have proposed correcting mitophagy and maintaining mitochondrial normal function as potential PD intervention means, which further indicates the necessity of research on mitochondria-related hypoxia.

Toxic gases in environmental pollution, such as CO and hydrogen sulfide (H_2_S), can act on mitochondrial complex IV and prevent cytochrome oxidase reduction in tissues, which can no longer carry out electron transfer, thus interrupting the respiratory chain and preventing biological oxidation. The oxidative phosphorylation process is affected by vitamin B1 and B2 deficiency caused by malnutrition. High temperature, radiation, and bacterial toxins damage mitochondria. Rotenone, paraquat, and MPTP are recognized as mitochondrial inhibitors (Millar et al., 2007). More immediately, pesticide poisoning itself can cause tissue hypoxia (Eddleston et al., 2002).

Mitochondrial damage is considered to be a characteristic feature of aging, and mitochondria are major targets of hypoxia and ischemic damage, which further contribute to the body's exposure to hypoxia (Ham and Raju, 2017). All these indicate the urgency of the study of hypoxia.

## α-Syn Pathology in PD

The etiology of PD is complex and there are many risk factors, but all of these risk factors can lead to the occurrence of α-syn pathology. As a protein widely expressed in the brain, α-syn has powerful physiological functions. In the disease state, α-syn undergoes a series of important transitions, such as post-translational modification, aggregation, and propagation, which drive PD progression.

### α-Syn

α-syn, encoded by the SCNA gene on chromosome 4, is a highly abundant protein composed of 140 amino acids and widely expressed in the brain (Srinivasan et al., 2021). Naturally occurring α-syn consists of three domains: (1) N-terminal lipids bind α-helices, (2) non-amyloid-beta component (NAC) domain, and (3) C-terminal domain rich in acidic residues. The special structure of the NAC region helps the protein to undergo the transition from α-helical conformation to β-pleated sheet, allowing it to polymerize into toxic oligomers (Zhang et al., 2018). Under normal conditions, α-syn exists in two forms, mostly in the form of a monomer, but it also exists in the form of a helically folded tetramer, which maintains a dynamic balance with the monomer (Bartels et al., 2011). Helically folded tetramer has a lower tendency to aggregate into fibrin and helps stabilize α-syn. Therefore, when the proportion of natural tetramers decreases due to genetic mutations and other reasons, it can also lead to disease.

α-syn has powerful physiological functions: maintaining synaptic function (Longhena et al., 2019), influencing neurotransmitters such as dopamine release (Abeliovich et al., 2000; Salmina et al., 2021), maintaining cell membrane homeostasis (Fusco et al., 2018), influencing microglial production and function (Booms and Coetzee, 2021), participating in lysosomal and mitochondrial activities (Tripathi and Chattopadhyay, 2019), and scavenging heavy metals (Harischandra et al., 2015). However, compared with its physiological function, its pathological function has attracted more attention and has been studied more widely, mainly because it plays an irreplaceable role in the pathogenesis of PD. Under pathological conditions, α-syn successively forms dimer, oligomer, fibrils, and LBs (Rosborough et al., 2017). It is believed that the formation of α-syn aggregates is mainly due to posttranslational modification of α-syn (Bell and Vendruscolo, 2021). Among them, phosphorylation is the most prominent (Anderson et al., 2006; Machiya et al., 2010), in addition to acetylation (Barrett and Timothy Greenamyre, 2015), ubiquitylation (Liu et al., 2021), glycosylation (Vicente Miranda et al., 2016), and CTD truncation (Izumi et al., 2016).

### α-Syn Modification and Aggregation in PD

There are many posttranslational modifications of α-syn, including phosphorylation, ubiquitination, glycosylation, phosphorylation, and acetylation. Among these, phosphorylation at the Ser129 site is considered to be the main modification that promotes α-syn aggregation and induces the α-syn pathology (Fujiwara et al., 2002). The percentage composition of phosphorylated alpha-synuclein (p-α-syn) in normal brain tissue is <4%. However, in autopsies of patients with PD, it was found that the majority of α-syn in LBs exists in the form of phosphorylated protein (up to 90%) (Hasegawa et al., 2002). This conclusion was also confirmed in animal models. P-α-syn has gradually become a protein that can indicate PD pathology (Hasegawa et al., 2002; Scudamore and Ciossek, 2018).

Phosphorylation of α-syn makes it easier to form aggregates, which are found in the nervous system of patients with PD, including the central nervous system and the peripheral nervous system (Bendor et al., 2013). In the central nervous system, α-syn aggregates were first identified in the olfactory bulb (OB) and dorsal motor nucleus, and subsequently in the pontine tegmentum, amygdala, and cortex (Braak et al., 2003). In the peripheral nervous system, α-syn aggregates were first found in the enteric nervous system (Wakabayashi et al., 1990).

It is currently believed that the most toxic form of α-syn is a soluble oligomer, which is associated with endoplasmic reticulum (ER) stress, mitochondrial defects, proteasome inhibition, more obvious inflammatory response, autophagy, and lysosome dysfunction, membrane damage, synaptic dysfunction, and other injury mechanisms (Winner et al., 2011; Bengoa-Vergniory et al., 2017).

### Pathological α-Syn Propagation in PD

Because of anatomical connectivity and cell-to-cell communication, α-syn fibrils serve as seeds that propagate in a prion-like manner between adjacent cells and anatomically connected brain regions (Goedert et al., 2010; Mao et al., 2016). This propagation is considered to be a key event in the progression of PD (Braak et al., 2003; Mehra et al., 2019). There are two views on the origin and direction of the propagation of pathological α-syn. The traditional view is that pathological α-syn originates in the central nervous system, spreads first in the brain, and then spreads to other sites. Another view is that pathological α-syn originates in the peripheral intestinal nervous system, first appearing in the intestine and then propagating retrograde along the vagus nerve, involving the central nervous system.

In a clinical study, normal embryonic midbrain neurons were transplanted into PD patients and LB deposition was found in previously healthy neurons during follow-up. This experiment demonstrates the importance of pathological α-syn cell-to-cell transmission in PD pathogenesis (Li et al., 2008). Aged α-syn transgenic mice express α-syn pathology. Brain homogenate proteins were prepared and injected into the striatum and neocortex of asymptomatic mice. The presence of pathological α-syn in the nerve axis from the OB to the spinal cord in mice was observed within 3 months of injection and accelerated the appearance of neurodegenerative disease. α-syn preformed fibrils (PFFs) are pathologic α-syn fibrils prepared *in vitro*. The formation of LBs and the onset of neurodegenerative disease were accelerated by the administration of PFFs into the brain (Luk et al., 2012).

Regarding the second view, Braak's hypothesis suggests that α-syn pathology can spread in a fixed manner from the gastrointestinal tract to the ventral midbrain *via* the vagus nerve. It then selectively kills dopaminergic neurons in the substantia nigra compact (SNc). Lebouvier et al. (2008) first discovered lesions similar to those observed in the brain in the intestines of living PD patients in 2008. Subsequent clinical studies based on a large sample size reported that the risk of PD diagnosis was significantly reduced in follow-up statistics after vagus nerve trunk resection, which supported Braak's hypothesis to some extent (Svensson et al., 2015). Based on the brain–gut transmission hypothesis, combined with the early intestinal disease epidemiology of PD patients and the pathological propagation mechanism of α-syn, pathological aggregation and retrograde transmission of α-syn may occur in the intestinal tract very early. It has been reported that α-syn accumulates in the stomach, duodenum, and colon in gastrointestinal (GI) biopsies of PD patients and healthy individuals (Shannon et al., 2012; Sánchez-Ferro et al., 2015). In order to simulate the gut–brain transmission of α-syn in PD proposed by Braak's hypothesis, Holmqvis and colleagues performed the first experimental validation in an animal model (Holmqvist et al., 2014). They demonstrated that pathological α-syn reached the dorsal motor nucleus of the vagus nerve in the brainstem in a time-dependent manner after injecting PFFs into the intestinal wall. They provided the first experimental evidence that pathological α-syn may first travel from the gut to the brain (Holmqvist et al., 2014), a conclusion subsequently confirmed by several studies (Kim et al., 2019; Ahn et al., 2020; Challis et al., 2020).

## Direct Evidence of Hypoxia and α-Syn Pathology

Above, we described the important risk factors for PD, including genes, environment, aging, and TBI. These factors all increase the expression or aggregation of α-syn, the key pathological protein of PD. At the same time, among these risk factors that can directly cause PD pathology, we found that all have oxygen intake or utilization disorders. Recent literature has confirmed the relationship between hypoxia and PD and reported that hypoxia regulation may be a key therapeutic target for PD (Burtscher et al., 2021). In the following sections, we review the effects of hypoxia on α-syn pathology from the perspectives of modification, aggregation, and propagation.

### Hypoxia Promotes α-Syn Modification and Aggregation

The phosphorylation and aggregation of α-syn are necessary steps in the pathogenesis of α-syn and PD development. The accumulation of misfolded and aggregated forms of α-syn increased under hypoxia (Muddapu and Chakravarthy, 2021). Clinically, patients with obstructive sleep apnea (OSA) directly face the problem of prolonged and repeated hypoxia (Sozer et al., 2018). Plasma levels of total α-syn and p-α-syn were significantly elevated and positively correlated with oxygen saturation (Sun et al., 2019). In animal model validation, α-syn expression increased and accumulated in a time-dependent manner by placing mice in a closed, wide-mouth bottle with limited oxygen to simulate hypoxia (Yu et al., 2004). The model of middle cerebral artery occlusion (MCAO) is characterized by ischemia and hypoxia injury. The expression of total α-syn and p-α-syn increased in rodent MCAO models (Unal-Cevik et al., 2011; Kim et al., 2016). Hypoxic ischemia was induced by ligation of the left common carotid artery in rats, and systemic hypoxia was induced by direct hypoxic treatment with 7.8% oxygen for 90 min. The expression of α-syn was found to be 2 times as high as before (Hu et al., 2006). Abnormal aggregation of α-syn induced by hypoxia and consequent neuronal apoptosis in the cerebral cortex has been observed in rats with acute alcohol intoxication (Li et al., 2016). In a cell model, total α-syn, p-α-syn, and their oligomers were increased by hypoxia treatment at 0.5% O_2_ for 24 h or hypoxia treatment at 1% O_2_ for 48 h. In addition, hypoxic treatment of cells with 1% O_2_ for 24 h increased α-syn expression but did not seem to lead to oligomer formation (Chen et al., 2014, 2019). These findings suggest that hypoxia may be an important factor in inducing the phosphorylation and aggregation of α-syn.

### Hypoxia Promotes α-Syn Propagation

Following phosphorylation and aggregation (Zhang et al., 2021), pathological α-syn can spread and propagate in a prion-like manner and is considered to be an important stage in the progressive pathogenesis of PD. The intestinal tract is considered to be the origin of α-syn pathology. Compared with other organs, the gastrointestinal tract is characterized by a steep oxygen gradient from the anaerobic lumen to the highly vascularized submucosa. Once intestinal inflammation occurs, the oxygen supply from the blood is reduced, and the resulting imbalance in oxygen consumption leads to more oxygen deprivation in the inflamed intestinal mucosa (Van Welden et al., 2017; Ananthakrishnan et al., 2018). Hypoxia and the hypoxia signaling pathway play an important role in the occurrence and development of intestinal diseases (Van Welden et al., 2017). Multiple population-based cohort studies have shown that patients with inflammatory bowel disease (IBD) have a higher risk of developing PD in later life, about 20–90% higher than the normal population. Chronic intestinal inflammation can also promote the progression of PD (Lin et al., 2016; Park et al., 2019; Weimers et al., 2019). In addition, pathological findings of IBD patients also revealed α-syn aggregates in the submucosa of the gastrointestinal tract (Prigent et al., 2019).

As mentioned above, hypoxia (Burtscher et al., 2021) and IBD (Park et al., 2019) have been reported to be related to PD, respectively, and the research value of hypoxia as a therapeutic target for PD has been confirmed. At the same time, it is clear that hypoxia and inflammation mutually promote each other. Tissue hypoxia, such as organ transplantation (Krüger et al., 2009), enlarged adipose tissue (Ye, 2009), and cancer (Semenza, 2003), leads to inflammatory changes. Inflammatory diseases such as colitis (Lv et al., 2018) and infections with pathogens (Devraj et al., 2017) often cause obvious tissue hypoxia. A growing number of studies have suggested that inflammation has been an important factor in promoting α-syn aggregation and transmission (Campos-Acuña et al., 2019; Resnikoff et al., 2019; La Vitola et al., 2021). There is also a lot of evidence that targeted inflammation can reduce PD presentation and delay its progression (Peter et al., 2018; Kishimoto et al., 2019). However, no specific studies have shown that hypoxia plays an important role in inflammation promoting abnormal protein aggregation. Given the importance of environmental stress factors to abnormal accumulation of α-syn and the complex reinforcing relationship between inflammation and hypoxia, hypoxia may play an important role in the formation and transmission of α-syn (Srivastava et al., 2020). However, further studies using more techniques, such as transgenic animal models, are needed to determine whether hypoxia plays a role as a promoter in various diseases such as inflammatory bowel disease.

## Limitations and Prospects

**Figure 1:**
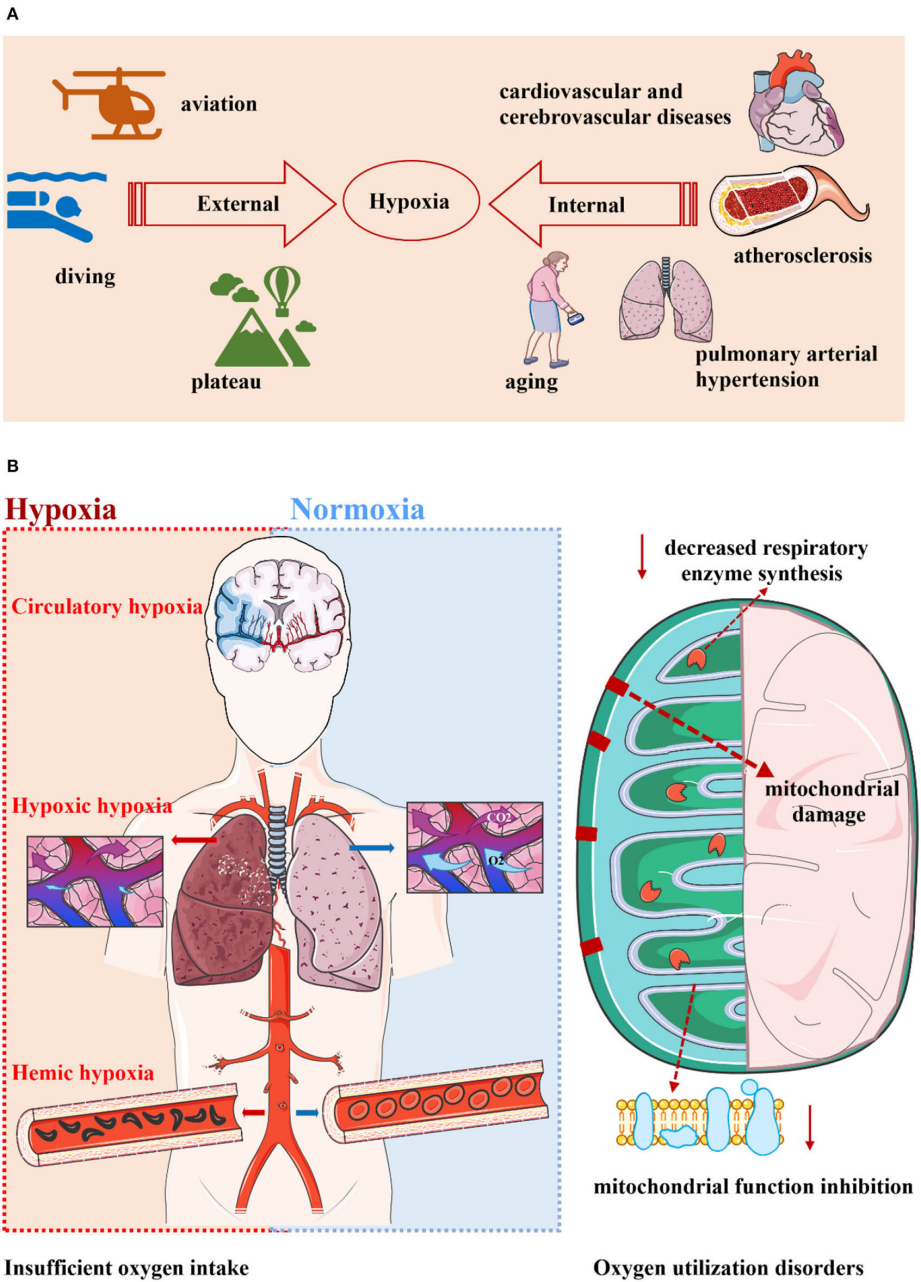
Hypoxia is widespread and can be divided into two categories. **(A)** Hypoxia is a very common phenomenon. Whether it is in the environment or in the body, hypoxia seems to indicate a decrease in oxygen levels that makes it difficult to sustain metabolism. Environmental hypoxia is common in the plateau, diving, and aviation. The pathological state of the body is accompanied by hypoxia, and diseases such as aging, cardiovascular and cerebrovascular diseases, pulmonary hypertension, and atherosclerosis all have pathological changes represented by hypoxia. **(B)** Hypoxia can occur in two ways: inadequate oxygen intake and oxygen utilization disorders. The former includes hypoxic hypoxia, hemic hypoxia, and circulatory hypoxia. The latter is known as tissue hypoxia, which is mainly related to mitochondrial damage, mitochondrial function inhibition, and reduced respiratory enzyme synthesis.

**Figure 2:**
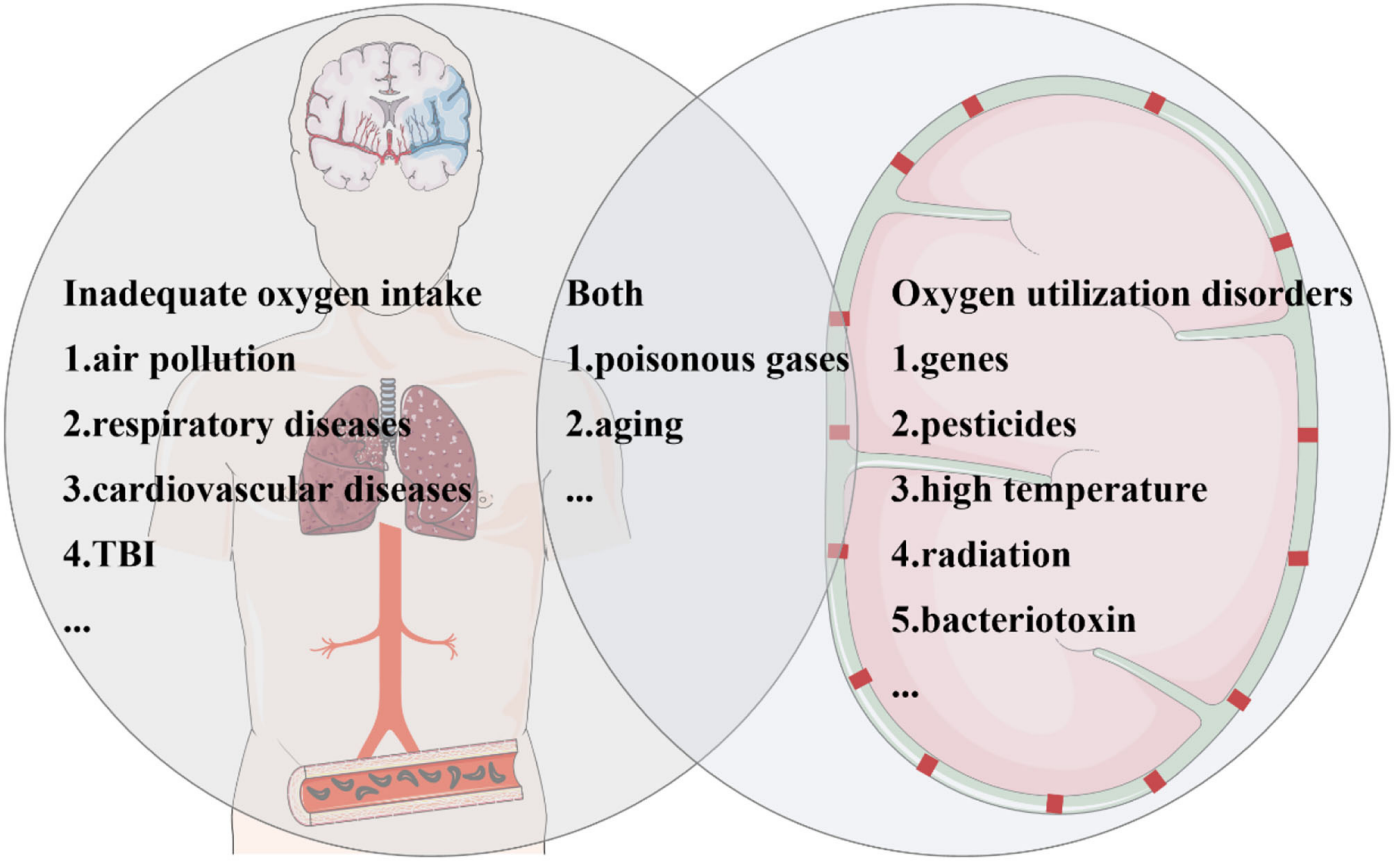
Parkinson's disease (PD) risk factors are closely related to hypoxia. Risk factors for PD associated with hypoxia can be divided into three categories. Some factors increase the risk of PD due to inadequate oxygen intake. Polluted air brings lower oxygen saturation, which can be caused by environmental particulates, automobile exhaust, and so on. The common pathological process of cardiovascular and cerebrovascular diseases and traumatic brain injury (TBI) is ischemia and hypoxia. Several factors contribute to oxygen utilization disorders, including genes, pesticides, high temperature, radiation, and bacterial toxins. In addition, aging, toxic gases, and carbon monoxide poisoning not only affect oxygen intake but also cause oxygen utilization disorders.

**Figure 3:**
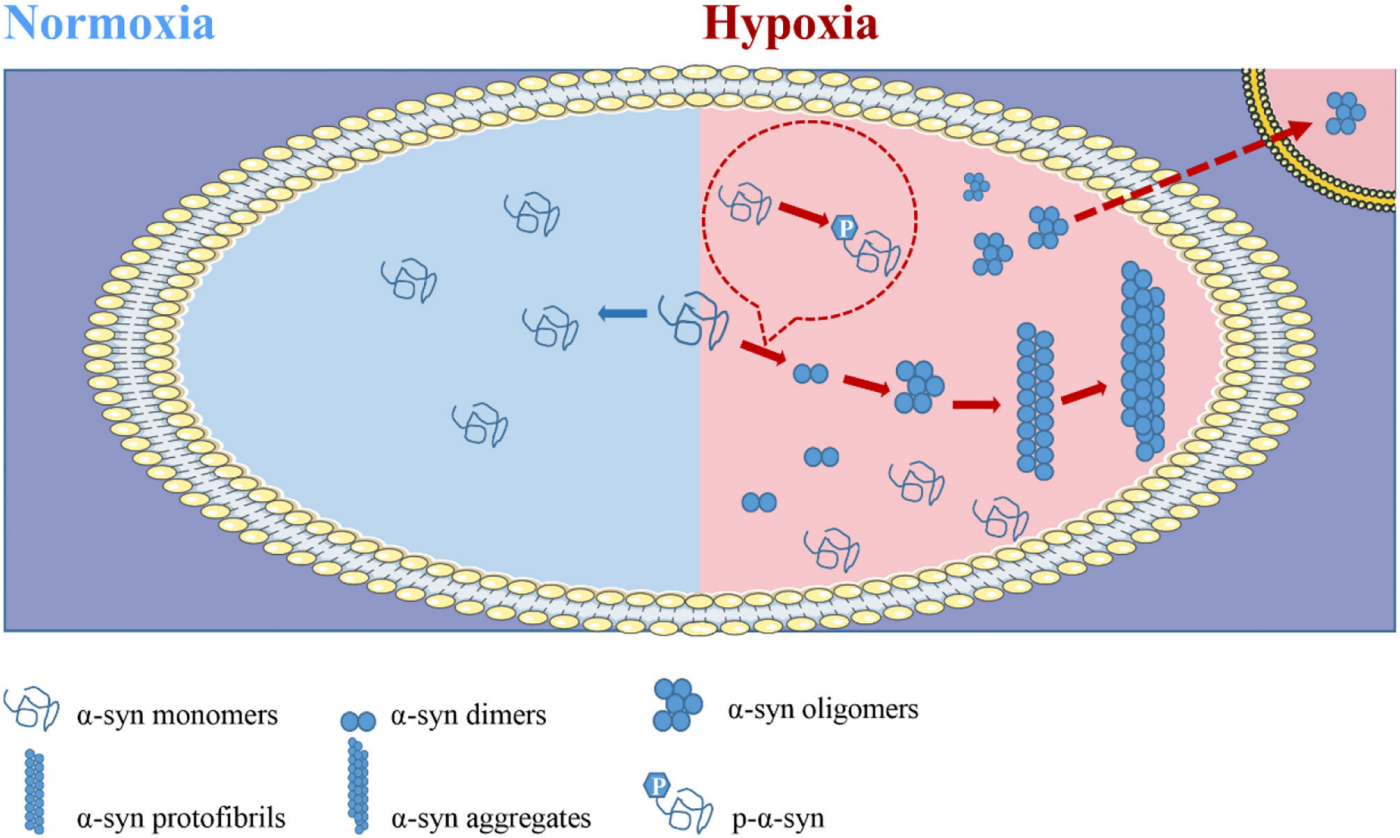
Hypoxia is involved in the pathogenesis of alpha-synuclein (α-syn) and promotes its propagation. In the natural state, the vast majority of α-syn exists in the form of a disordered monomer, and almost no modification forms or aggregates in healthy machines. In conditions such as PD, α-syn is present in aggregates. α-syn promotes disease progression by aggregation and propagation. *In vivo*, α-syn successively forms dimers, oligomers, fibrils, and aggregates, and it eventually forms LB sediments. The oligomers act as seeds to infect healthy cells and spread the disease. Importantly, the formation of such pathological aggregates is dependent on posttranslational modification, especially phosphorylation. Hypoxic exposure is most likely to occur when the body is in a state of disease or for external reasons. Current studies suggest that hypoxia promotes the modification, aggregation, and transmission of α-syn, and thus promotes disease progression.

A large number of patients suffer from PD, and the current treatments only focus on the use of dopaminergic drugs to control PD symptoms. Such treatments cannot fundamentally solve the problem or even alleviate the huge mental and economic burden brought by PD. Therefore, it is necessary to explore the pathogenesis of PD in depth and to find possible intervention targets from the early stages of PD. An exploration of this sort will provide new ideas for slowing or stopping the progression of the disease. However, in view of the complex etiology of PD, the numerous influencing factors, and the unknown pathogenesis, it is particularly important to find common ground in the many links of PD progression. In the pathogenesis of PD, the modification, aggregation, and propagation of key pathological protein α-syn are closely related to the development of PD. As noted above, multiple PD risk factors have been shown to be associated with hypoxia (Figures 1, 2), and several studies have confirmed that the hypoxic stress promotes the phosphorylation and aggregation of α-syn (Figure 3). The abnormal aggregation and accumulation of α-syn are affected by many factors. Despite the abnormal increased α-syn level promoting its abnormal aggregation, there are also various factors in α-syn pathology. Risk genes expression, disrupted cellular microenvironment, impaired membrane interaction, increased polyamines, increased charged polymers, and abnormal modifications also contribute to the aggregation and accumulation of α-syn. As mentioned above, hypoxia may trigger, facilitate and aggravate the abnormal pathology of α-syn by modulating the above factors, but the underlying mechanism is unclear.

In spite of this, we regret that there are no clear studies showing that hypoxia has a more profound effect on PD than other neurodegenerative diseases. Only a few studies have reported that hypoxia may be more closely related to PD (Burtscher et al., 2021). This conclusion of the above article may be partly related to this reason: a search of current studies using HIF-1α activators or inhibitors to intervene in the ultimate pathology of the disease revealed a preponderance of PD-related articles. Among them, most of the studies are based on the treatment strategies for PD based on the activation of HIF-1α, involving indirect PHD inhibitors, competitive PHD inhibitors, and atypical HIF-1α inducers (Lestón Pinilla et al., 2021). As for which neurodegenerative diseases are more affected by hypoxia and its proportion in pathogenesis, more detailed research are still needed. The association between PD and hypoxia may also be explored through the assumption that SNc dopaminergic neurons are more susceptible to hypoxia. SNc dopaminergic neurons have higher energy metabolism requirements, and their basal respiration level is about 3 times higher than that of dopaminergic neurons in other regions such as VTA or OB. In addition, SNc dopaminergic neurons have lower respiratory reserve capacity, higher basal glycolysis level, larger axonal arborization, the higher mitochondrial density of mitochondria, and greater vulnerability to cytotoxins (Pacelli et al., 2015). In addition, HIF-1α is associated with the development and survival of SNc dopaminergic neurons (Milosevic et al., 2007), and the increased expression of key proteins such as Tyrosine hydroxylase (TH), DA transporter (DAT) (Lim et al., 2015). The above factors may lead to the vulnerability of SNc dopaminergic neurons and a certain degree of hypoxia susceptibility. Meanwhile, in view of the typical pathological characteristics of PD is the progressive loss of dopaminergic neurons in the SNc, previous studies focused on more vulnerable neurons. At present, more and more studies have proved that PD-related genes such as SNCA, PARK2, and PINK1 may be expressed in glia including microglia (Miklossy et al., 2006) and astrocytes (Booth et al., 2017). Glial cells may play a driving role in the pathogenesis and progression of PD through homeostasis imbalance, dysfunction, and neurotoxicity (Kam et al., 2020). But the exact mechanism is unclear. There is also evidence that hypoxia modulates HIF-1α in microglia and induces microglial autophagy (Yang et al., 2014). However, the role of glial cells under hypoxia in PD pathology and pathogenesis has not been determined, which is a very important research direction and needs further exploration in the future.

Moreover, there is still a lack of adequate attention and systematic research in this field, and there are many pending problems: (1) the effect of hypoxia on α-syn pathological propagation remains unclear; (2) it is unknown whether differences in the degree, duration, and pattern of hypoxia make a difference in outcome; and (3) it remains to be determined whether it is possible to intervene in PD by resisting hypoxia or improving hypoxia tolerance. To sum up, it is a promising direction to explore the role of hypoxia in PD caused by different inducements in an in-depth and systematic way and find its common mechanism from a new perspective.

## Data Availability Statement

The original contributions presented in the study are included in the article/supplementary material, further inquiries can be directed to the corresponding author/s.

## Author Contributions

MG: visualization, investigation, and writing—original draft preparation. JL: conceptualization, visualization, and writing—reviewing and editing. XJ: conceptualization, resources, and supervision. All authors contributed to the article and approved the submitted version.

## Funding

This research was supported by the National Natural Science Foundation of China (Grant numbers: 32100925 and 82027802), the Beijing Nova Program (Grant number: Z211100002121038), the Beijing Hundred Thousand and Ten Thousand Talents Project (Grant number: 2019A36), and the Beijing Municipal Health Commission (Grant number: 303–01–005-0019).

## Conflict of Interest

The authors declare that the research was conducted in the absence of any commercial or financial relationships that could be construed as a potential conflict of interest. The reviewers SY and WY declared a shared affiliation with the authors XJ, MG, and JL to the handling editor at the time of the review.

## Publisher's Note

All claims expressed in this article are solely those of the authors and do not necessarily represent those of their affiliated organizations, or those of the publisher, the editors and the reviewers. Any product that may be evaluated in this article, or claim that may be made by its manufacturer, is not guaranteed or endorsed by the publisher.
